# Inferring the expression variability of human transposable element-derived exons by linear model analysis of deep RNA sequencing data

**DOI:** 10.1186/1471-2164-14-584

**Published:** 2013-08-28

**Authors:** Wensheng Zhang, Andrea Edwards, Wei Fan, Zhide Fang, Prescott Deininger, Kun Zhang

**Affiliations:** 1 Department of Computer Science, Xavier University of Louisiana, 1 Drexel Drive, New Orleans, LA 70125, USA; 2 Huawei Noah Ark’s Lab, Hong Kong, China; 3 Biostatistics Program, School of Public Health, Louisiana State University Health Sciences Center, New Orleans, Louisiana, USA; 4 Tulane Cancer Center, Tulane School of Public Health and Tropical Medicine, New Orleans, Louisiana 70122, USA

## Abstract

**Background:**

The exonization of transposable elements (TEs) has proven to be a significant mechanism for the creation of novel exons. Existing knowledge of the retention patterns of TE exons in mRNAs were mainly established by the analysis of Expressed Sequence Tag (EST) data and microarray data.

**Results:**

This study seeks to validate and extend previous studies on the expression of TE exons by an integrative statistical analysis of high throughput RNA sequencing data. We collected 26 RNA-seq datasets spanning multiple tissues and cancer types. The exon-level digital expressions (indicating retention rates in mRNAs) were quantified by a double normalized measure, called the rescaled RPKM (Reads Per Kilobase of exon model per Million mapped reads). We analyzed the distribution profiles and the variability (across samples and between tissue/disease groups) of TE exon expressions, and compared them with those of other constitutive or cassette exons. We inferred the effects of four genomic factors, including the location, length, cognate TE family and TE nucleotide proportion (RTE, see Methods section) of a TE exon, on the exons’ expression level and expression variability. We also investigated the biological implications of an assembly of highly-expressed TE exons.

**Conclusion:**

Our analysis confirmed prior studies from the following four aspects. First, with relatively high expression variability, most TE exons in mRNAs, especially those without exact counterparts in the UCSC RefSeq (Reference Sequence) gene tables, demonstrate low but still detectable expression levels in most tissue samples. Second, the TE exons in coding DNA sequences (CDSs) are less highly expressed than those in 3′ (5′) untranslated regions (UTRs). Third, the exons derived from chronologically ancient repeat elements, such as MIRs, tend to be highly expressed in comparison with those derived from younger TEs. Fourth, the previously observed negative relationship between the lengths of exons and the inclusion levels in transcripts is also true for exonized TEs. Furthermore, our study resulted in several novel findings. They include: (1) for the TE exons with non-zero expression and as shown in most of the studied biological samples, a high TE nucleotide proportion leads to their lower retention rates in mRNAs; (2) the considered genomic features (i.e. a continuous variable such as the exon length or a category indicator such as 3′UTR) influence the expression level and the expression variability (CV) of TE exons in an inverse manner; (3) not only the exons derived from Alu elements but also the exons from the TEs of other families were preferentially established in zinc finger (ZNF) genes.

## Background

Recent years have witnessed the emergence of a few high throughput technologies, i.e. Affymetrix exon array and RNA-seq platforms, for generating large-scale exon-level gene expression profiling. While array-based data have been widely employed to study transcript splicing [1-3], there are inherent limitations of this technique, such as the potential cross-hybridization of the probes of one exon to the transcript of another exon, and the incomplete probe coverage [3,4]. Especially, because the probe design for a gene is based on the exons included in one or multiple manually annotated or computationally predicted gene models [5], many infrequently used exons are never represented by any probe, and therefore their expression levels cannot be measured accordingly. This problem can be circumvented by RNA-seq technology, which provides hypothesis-free single nucleotide resolution of gene expression so that, theoretically, any expressed sequence can be detected and quantified, given appropriate computational/statistical methods and sufficient sequencing depth. RNA sequencing based gene expression data have become the major information source for detecting alternative splicing (AS), alternative cleavage, polyadenylation (APA) events, gene fusions, and splicing eQTLs (expression Quantitative Trait Loci) [4,6-11].

Transposable elements (TEs) constitute approximately 44% of the human genome. In disease biology, the importance of TEs is highlighted by the potential association with genetic instability, one of the principal hallmarks and causative factors in cancer [12-15]. A recent study showed that Estrogen Receptor α (ERα), which is involved in human breast cancer, preferentially targets mammalian interspersed repeats (MIRs) transposons [16]. The exonization of mutated TE sequences has proven to be a significant mechanism for the creation of novel exons [17-20]. In the TranspoGene database, 1423 human exonized TEs (TE exons), involved in ~1700 RefSeq genes, have been collected [21]. Most TE exons are alternatively spliced during the post-transcriptional modification of RNAs [22]. Nevertheless, they are not infrequent in mature transcripts [4,21,23]. Past studies have suggested the diverse roles of TE alternative splicing in gene regulation [24-27]. Many human genetic diseases have been ascribed to TE exonization [28-30]. Analysis of single nucleotide polymorphisms (SNPs) also revealed that exonization of TEs can be population-specific, implying that exonizations may enhance divergence and lead to speciation [20].

The retention and splicing of a TE exon has been found related to multiple genomic factors, such as the TE category and exon length [21,31]. These published results were mainly established by the analysis of EST and microarray data using descriptive statistical methods, where other factors cannot be considered simultaneously when inferring the effect of a specific one. In this work, we extended the previous studies by an integrative statistical analysis of multiple RNA sequencing data. In particular, we not only studied the digital expression levels of TE exons but also addressed the variability quantities of TE exon expressions across different biological samples and groups. Our findings provide advanced insights into the establishment level of TE exons and their roles in the evolution of human genome.

## Results

We collected 26 published RNA-seq datasets spanning multiple tissues and cancer types [9,32-36]. Each of them has at least 30 million Illumina sequence reads of 33-75 bp and is from a genetically or pathologically specific sample. An exception is the dataset for liver tissue, which was generated by combining six subsets of different individuals. The gene- and exon-level digital expression is inferred by the approach presented in the Methods section. The details of sample collection and reads mapping are summarized in Table 1.

**Table 1:** **Summary of the analyzed samples and datasets**^**$**^

**Sample ID**	**Sample description**	**Platform**	**Mapped reads**	**Filtered reads**	**Resources**
BT20	ER- breast cancer cell line	P/50	137897876	118362274	GEO: GSE27003; Sun et al., 2011 [35]
MDAMB231	ER- breast cancer cell line	P/50	96483262	80759008	Same as above
MDAMB468	ER- breast cancer cell line	P/50	123622490	104468212	Same as above
MCF7	ER + breast cancer cell line	P/50	129592066	107422464	Same as above
BT474	ER + breast cancer cell line	P/50	131597078	110877774	Same as above
T47D	ER + breast cancer cell line	P/50	119708904	99784684	Same as above
ZR751	ER + breast cancer cell line	P/50	107891488	90362316	Same as above
MCF10A	Normal breast cell line	P/50	125148556	108184998	Same as above
LNCaP	Prostate cancer cell line	S/35	38953595	27126677	GEO: GSE29155; Kim et al., 2011 [33]
PrEC	Normal prostate cell line	P/35	27825207	22366081	Same as above
LCL-1^a^	Lymphocyte cell lines	P/36,38	64168681	54301769	GEO: GSE25030; Montgomery et al., 2010 [34]
LCL-2	Lymphocyte cell lines	P/36,38	71571009	60017609	Same as above
OV-1-pr^b^	Ovarian cancer cell line	P/42	75756477	69739708	SRA: ERP000710 [36]
OV-1-re	Ovarian cancer cell line	P/42	95813480	89853848	Same as above
OV-2-fi	Ovarian cancer cell line	P/42	90793509	84057374	Same as above
OV-2-se	Ovarian cancer cell line	P/42	100491160	93088808	Same as above
OV-3-pr	Ovarian cancer cell line	P/42	72611523	66647818	Same as above
OV-3-re	Ovarian cancer cell line	P/42	89393849	84067652	Same as above
prAd_1^c^	Prostate adenocarcinoma	S/33	32495059	23386861	GEO: GSE24283; Nacu et al., 2011 [9]
prAd_2	Prostate adenocarcinoma	S/33	34663805	24398162	Same as above
prAd_3	Prostate adenocarcinoma	S/33	66976637	48613865	Same as above
prNorm_1	Normal prostate tissue	S/50,75	37201269	30394802	Same as above
prNorm_2	Normal prostate tissue	S/50	37451643	30392995	Same as above
prNorm_3	Normal prostate tissue	S/33	33511439	25294452	Same as above
Brain	Brain tissue	S/50	48741218	42660318	Same as above
Liver^d^	Liver tissue	S/35	31258238	26253587	GEO: GSE17274; Blekhman et al., 2010 [32]

^$^The letters and numbers in the third column represent sequencing types, single read (S) or paired-end reads (P), and read lengths, respectively. We excluded the non-primary hits when counting the mapped reads (fourth column). The numbers of unambiguously mapped reads are listed in the column of filtered reads.

^a^LCL-1 and −2 are Coriell human lymphocyte lines NA12892 and NA19238.

^b^-1, -2 and −3 indicate the IDs of the cell lines. –pr, -re, -fi, and –se indicate the clinical history. They represent “present”, “relapse”, “first relapse” and “second relapse”, respectively.

^c^-1, -2 and −3 are the IDs of prostate adenocarcinoma (or normal prostate tissue) samples.

^d^The data sets of 12 liver samples (replicates) were combined before we mapped reads to the human genome and the computationally identified exon-exon junctions.

### Expression of TE exons and the comparison with constitutive and cassette exons

By integrating the UCSC RefSeq annotation for human genome version 19 (hg19) [37] and the human TE exon information collected in the TranspoGene database [21], we established four exon classes (C1-C4). C1 contains 207498 non-TE-derived constitutive exons. C2 contains 42457 non-TE-derived cassette exons. C3 contains 215 annotated TE-derived exons. C4 contains 1191 previously un-annotated TE-derived exons. We regarded an exon as a “TE-derived” or “non-TE-derived” exon if it is present in or absent from the TranspoGene human exonized TEs table. We categorized a non-TE-derived exon as a “cassette” or “constitutive” exon if it is included or isn’t included in the UCSC known Alt table [21]. We labeled a TE exon to be “annotated” or “un-annotated” if it has or doesn’t have an exact counterpart with the same coordinates (i.e. the starting and ending positions in the genome) in the UCSC RefGene table. Furthermore, we simulated a fictional exon class (C5), which consists of 1191 intronic sequences immediately downstream of the C4 exons with the lengths equal to the corresponding C4 exons. For each class, we calculated the sample-specific ratios of un-expressed exons, and the mean and standard deviation of expression levels for the exons with minimum post-transcriptional retention (i.e., at least one read was mapped on the genomic region). The expression level was quantified by a double normalized measure (see Methods section), called the rescaled RPKM. This measure can well represent the inclusion level, which can’t be directly evaluated by the primary RPKM, of an exon in the transcripts coded by the host gene. Before the computation, we filtered out the exons in the “un-expressed” genes whose RPKMs were below a sample-specific cutoff. The cutoffs were determined by the RPKMs of 500 fictional genes, each of which is composed of 4–40 simulated exons in C5. Hereafter, we conducted all statistical analysis on the exons in the “expressed” genes and abbreviated the class names of C1 and C2 as “constitutive exons” and “cassette exons”, respectively.

As shown in Table 2, the decreasing trend of the expression levels and the increasing trend of the un-expressed exon ratios, in the order of C1, C2, C3, C4 and C5, are apparent and consistent across the 26 samples. We herein conducted a set of stepwise statistical tests. That is, we determined the significance levels of four comparisons, i.e. C1 versus C2, C2 versus C3, C3 versus C4 and C4 versus C5, by performing a Mann–Whitney test (for the first three comparisons of independent groups) or a Wilcoxon signed-rank test (for C4 versus C5, a comparison of paired groups). The corresponding p-values are listed in the columns 4, 7, 10 and 13 of Table 2, respectively. As expected, cassette exons (C2) are less expressed compared to constitutive exons (C1) (p < < 4.33e-26). Annotated TE exons (C3) contain both constitutive exons (30%) and non-constitutive exons (70%), but the expression levels on average are less than half of the cassette exons’ (C2) expression. Typically, 17-60% of the exons in this class are un-expressed with respect to different samples. The maximum p-value for C3 versus C2 comparisons is 1.82e-14. The expressions level of C3 exons is significant higher than that of the previously un-annotated TE exons (C4) with the maximum p-value less than 1.19e-6. Considering that ETS databases represent a main information resource for gene annotation, it seems certain that frequently-expressed TE exons are more likely included in populous transcripts than the less-expressed ones (we assume that the inclusion of a transcript in RefSeq table indicates its popularity). However, our main interest is whether, as a whole, the un-annotated TE exons have minimum expressions in most samples. The statistical analysis indicates that the expression signals of these TE exons are detectable. In the comparison between the un-annotated TE exons (C4) and the simulated exons (C5), 20 of the 26 samples have a p-value less than 0.01, indicating that the un-annotated TE exons (C4) are expressed at modestly higher and significant levels in most tissues. Here, the C5 expression represents our best estimate of background levels of sequences that may come from DNA contamination or the presence of unprocessed nuclear RNAs.

**Table 2:** Statistical analysis on the digital expressions of the exons in different classes

**Sample**	**Constitutive exons**			**Cassette exons**			**Annotated TE exons**			**Un-annotated TE exons**		
	**R**	**M**	**p**	**R**	**M**	**p**	**R**	**M**	**p**	**R**	**M**	**p**
BT20	0.03	0.98	1.72E-36	0.07	0.91	3.11E-29	0.26	0.38	4.28E-20	0.51	0.07	7 7.43E-20
MDAMB231	0.05	0.98	1.89E-49	0.09	0.9	4.67E-28	0.33	0.42	2.20E-19	0.6	0.07	7.65E-16
MDAMB468	0.04	0.99	4.64E-69	0.08	0.9	9.92E-33	0.28	0.36	8.99E-23	0.58	0.07	3.54E-12
MCF7	0.03	0.99	8.91E-64	0.07	0.89	1.75E-34	0.3	0.31	2.54E-12	0.48	0.07	1.45E-20
BT474	0.03	0.99	3.81E-62	0.08	0.9	4.31E-34	0.36	0.35	6.87E-13	0.53	0.07	1.22E-12
T47D	0.04	0.98	4.33E-26	0.08	0.92	5.72E-34	0.2	0.35	3.00E-31	0.58	0.06	7.08E-16
ZR751	0.04	0.99	7.93E-58	0.08	0.9	1.06E-31	0.29	0.3	3.26E-19	0.6	0.07	6.88E-10
MCF10A	0.04	0.99	2.00E-72	0.09	0.89	2.26E-31	0.28	0.36	8.59E-26	0.6	0.06	4.63E-14
LNCaP	0.09	0.99	4.16E-91	0.16	0.88	6.58E-23	0.39	0.38	2.99E-21	0.7	0.08	4.12E-05
PrEC	0.08	0.99	1.18E-121	0.15	0.86	7.20E-28	0.49	0.33	2.55E-11	0.7	0.08	6.84E-05
LCLs	0.02	0.99	2.92E-136	0.06	0.86	9.98E-35	0.18	0.3	3.05E-24	0.45	0.06	1.00E-15
OV-1-pr	0.02	0.98	9.29E-50	0.05	0.91	4.59E-43	0.23	0.29	3.65E-15	0.39	0.08	1.17E-07
OV-1-re	0.03	1	2.53E-188	0.06	0.84	1.32E-36	0.17	0.3	6.77E-19	0.39	0.08	3.36E-06
OV-2-fi	0.02	0.99	1.29E-153	0.05	0.86	3.86E-35	0.17	0.34	6.15E-16	0.29	0.11	8.54E-07
OV-2-se	0.02	0.99	8.43E-77	0.04	0.89	6.42E-46	0.2	0.3	2.13E-13	0.31	0.09	4.55E-07
OV-3-pr	0.03	0.99	2.01E-162	0.07	0.84	7.23E-42	0.35	0.23	2.60E-08	0.47	0.1	1.19E-01
OV-3-re	0.02	0.99	2.39E-109	0.05	0.87	1.84E-40	0.17	0.27	1.51E-16	0.4	0.08	4.65E-07
prAd_1	0.14	1	1.24E-256	0.25	0.71	1.82E-14	0.57	0.28	2.43E-12	0.81	0.05	2.42E-01
prAd_2	0.12	1.01	7.82E-292	0.22	0.72	6.88E-17	0.6	0.3	7.12E-10	0.79	0.05	2.50E-01
prAd_3	0.07	1.02	0.00E + 00	0.16	0.71	3.15E-19	0.46	0.27	7.33E-13	0.7	0.05	1.83E-02
prNorm_1	0.07	0.99	7.83E-170	0.13	0.83	2.49E-34	0.42	0.3	1.35E-17	0.68	0.06	2.23E-06
prNorm_2	0.07	0.99	1.41E-172	0.12	0.83	2.33E-26	0.4	0.33	1.39E-14	0.65	0.07	2.01E-05
prNorm_3	0.13	1.01	5.47E-288	0.23	0.74	7.63E-16	0.55	0.35	3.49E-12	0.78	0.06	7.63E-02
Brain	0.04	0.99	6.99E-183	0.09	0.84	5.03E-26	0.31	0.31	1.70E-13	0.54	0.08	1.30E-09
Liver	0.08	1	8.84E-124	0.15	0.85	5.08E-28	0.52	0.26	1.19E-06	0.68	0.09	1.67E-01

**R**: the proportion of exons with no reads mapped to the genomic regions in the alignment among the entire set of exons in the corresponding categories. **M**: the average of the rescaled RPKMs. **p**: the p-values for stepwise comparisons (see Results section for details).

**Figure 1:**
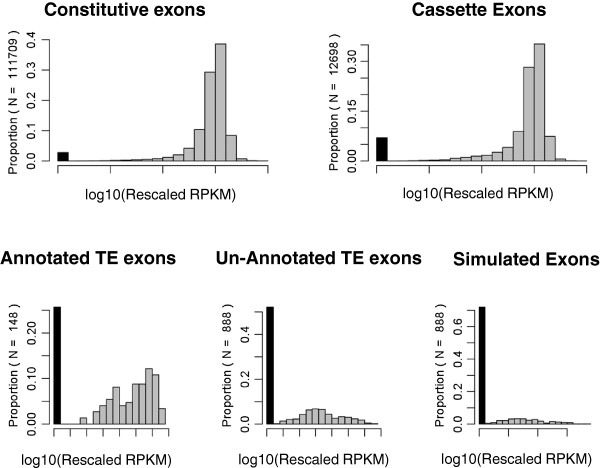
**Histograms for the digital expression levels of TE exons in sample BT20 (representing cluster G1).** The black bar on the left side of each plot represents the proportion of un-expressed exons.

We visualized the sample-specific distribution of exon expression levels in each class (C1-C5) by the histograms of log10 transformed rescaled RPKMs. Based on the visualization (mainly on the profiles for C4. See Figure 1, Additional files 1 and 2), we divided the 26 samples into three clusters with respect to the relative levels of expression. Of the 26 samples, 16 (62%) are in the first cluster (G1). They include seven breast cancer lines (BT20, MDAMB231, MDAMB468, MCF7, BT474, T47D, ZR751); one normal breast cell line (MCF10A); one prostate cancer cell line (LNCaP); one normal prostate cell line (PrEC); two lymphocyte cell lines (LCL-1, 2); two normal prostate tissue samples (prNorm-1, 2); one brain tissue sample, and the combined liver sample. As demonstrated in Figure 1, the representative plot for cluster G1, a part of un-annotated TE exons have apparent expression signals in these samples and the distribution profiles are distinguishable from the counterparts of the simulated exons. The second cluster (G2) includes all the three prostate adenocarcinoma samples (prAd-1, 2, 3) and one normal prostate sample (prNorm-3), where the expression signals of un-annotate TE exons are very weak (Additional file 1). The last cluster (G3) includes six samples (OV-1-pr, OV-1-re, OV-2-fi, OV-2-se, OV-3-pr, and OV-3-re) of three ovarian tumor cell lines at different pathological stages, where the expression signals of un-annotated TE exons are strong. However, the reads mapped on the sequences of the simulated exons are numerous (Additional file 2), indicating substantial existence of pre-RNAs in those samples.

In light of the apparent association between the expression-based sample grouping (G1, G2 and G3) and the tissue/disease type-based classification, a tissue specific mechanism in gene expression possibly underlies the differences among G1, G2 and G3 in the profiles of the retention rates of TE exons. However, this observation is subject to the potential statistical confusion caused by the tissue (disease) type effects (if existed) and the experiment-related batch effects in the analyzed data. Moreover, the speculated tissue (disease) type effects warrant further validation with additional data. Other explanations to the observed association are also explored in the Discussion section.

### Cross-sample variability of the TE exon expressions

**Figure 2:**
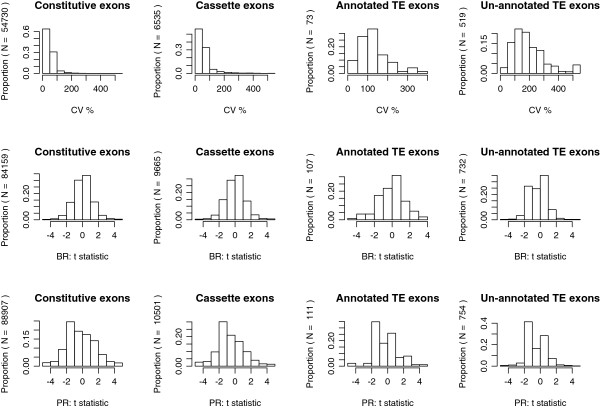
**Histograms for the variability and standardized inter-class differences of the digital expression levels of TE exons.** CV: the coefficient of variance of the rescaled RPKMs across the 26 samples. BR t-statistic: calculated with the difference and pooled standard deviation of the rescaled RPKMs for three ER- breast cancer cell lines and four ER + breast cancer cell lines. PR t-statistic: calculated with the difference and pooled standard deviation of the rescaled RPKMs for three prostate adenocarcinoma samples and three normal prostate tissue samples.

The statistical analysis presented above suggested the high variability of TE exon retention in transcripts. This perception can be further strengthened by comparing the cross-sample variability of the rescaled RPKMs for exons in different classes (C1-C4). Firstly, we calculated the coefficients of variance (CVs) for the exons whose host genes were expressed in at least half of the samples. The results showed that the CVs of TE exons were abnormally high with most values falling in the interval 50% ─ 400% (Figure 2). In contrast, although the un-expressed ratios of cassette exons were at least one time higher than those of constitutive exons (Table 2), the CV distribution profiles of both exon classes were largely in the same shape. Then, we calculated the t-statistics for two comparisons: (1) estrogen receptor-positive (ER+) versus estrogen receptor-negative (ER-) subtypes of breast cancer cell lines, and (2) prostate adenocarcinoma versus normal prostate tissue. As shown in Figure 2, the distribution profiles of the t-statistics in TE exon classes, especially in the un-annotated TE classes, are different between these two comparisons. The shape demonstrated a left-skewed single-mode pattern in (1) but resembled a bimodal distribution in (2). In addition, using the expression quantities of these exons, we can largely separate the three prostate tumors from their adjacent normal tissue samples by a complete-linkage hierarchical clustering algorithm with Euclidean distance measure. We attribute this observation to two potential reasons. First, the disease process in the tumor samples might influence the alternative splicing of the transcripts of TE exon host genes. Second, there was a (not-recorded technical) batch effect in generating the short read sequencing data for these tumor and normal prostate samples.

### Linear model analysis of the relationship between genomic factors and TE exon expressions

Based on the TranspoGene/Human exonized TEs table and a preliminary analysis, we determined four categorical or numerical genomic factors with each individually associated with the expression levels of TE exons. Then, we stringently inferred the effects of these factors on the dependent variables (indicating or measuring TE exon expression) by a set of linear models. Four factors were considered as the independent variables (predictors). They include the location (CDS, 3′UTR, and 5′UTR) of a TE exon in the host gene; the TE family (Alu, L1, etc.) to which the exonized TE belongs; the TE exon length (ELN); and the TE nucleotide proportion (RTE, see Methods section for the calculation) of a TE exon.

**Figure 3:**
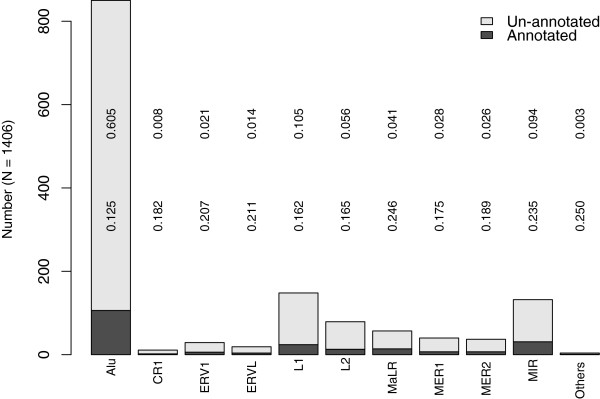
**Distribution of TE exons by the cognate TE families and the inclusion (presence or absence) in the UCSC RefGene table.** A number in the upper row is the proportion of the un-annotated TE exons within the corresponding family among the entire set. A number in the lower row is the proportion of the annotated TE exons within a family.

We begin the linear model analysis by a brief description of the two categorical factors. TE exons located in CDSs or in both UTR and CDSs, amount to 66.7% of the entire TE exon set. 1.4% and 31.9% of TE exons are solely located in 3′UTRs and 5′UTRs, respectively. The classification of TE exon with respect to the cognate TE families is summarized in Figure 3. The exons derived from “CR1” and “other DNA” are rare and, therefore, are excluded from further analysis.

Considering the sparseness of TE exon retention in transcripts, similar to [15], we adopted a two-step approach in the analysis. The first step (by Model-1) evaluates the effects of the quantitative predictors (ELN and RTE) and categorical predictors (location in the host gene and TE family) on the presence or absence of TE exons in the transcripts of their host genes. The second step (by Model-2) evaluates the effects of the same quantitative and categorical predictors on the rescaled RPKMs of the TE exons with non-zero expression. The exons within the un-expressed genes were excluded from the analysis. Log10 transformation was applied to the dependent variable of Model-2.

**Figure 4:**
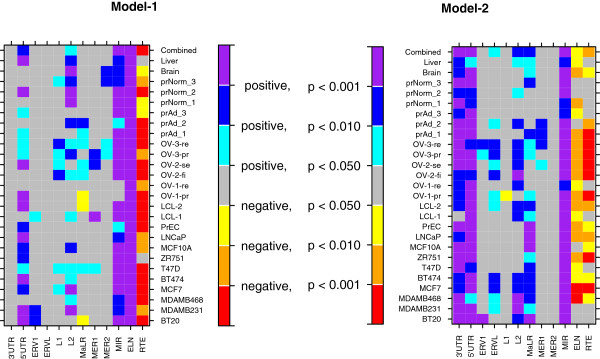
**Visualization of the effects of genomic factors on the digital expression of TE exons.** The results indicated by the top rows of color boxes are inferred by the median of TE exons’ un-expressed ratio and rescaled RPKMs across all samples. In the analysis, Alu and CDS are set as the baselines for the two categorical factors, location and TE family, respectively.

We summarized the sample-specific results in Figure 4, where the discretized p-values were presented for the corresponding effects. For the first categorical factor (location in the host gene), the effects of 3′UTR and 5′UTR were evaluated with CDS as the baseline. For the second categorical factor, the effects of L1 and other seven TE families were evaluated with Alu as the baseline. Consistently across all the 26 samples, the TE exons in 5′UTRs and derived from MIR elements are more frequently included in transcripts (according to the Model-1′s result), and have higher expression levels (according to the Model-2′s result) compared to the baselines (CDS and Alu) with p < 0.01. A similar retention preference is shown among TE exons in 3′UTRs. However, due to the small exon subset, the class effect is not significant in Model-1 (p & 0.05). Besides MIR, L2 is another family of TE elements from which the (highly) expressed exons were commonly derived. The positive class effect is significant (p < 0.05) in both statistical models and for most samples. The third TE family related to the highly expressed TE exons is MaLR, where the class effect is significant in Model-2 for most samples. The TE nucleotide proportion in an exon negatively influences its retention in transcripts. For most samples, the negative RTE effect is significant (p < 0.05). The impact of the TE exon length (ELN) on the expression of TE exons is somewhat elusive. The result of Model-1 indicates that the expression evidence is more frequently observed among those exons of relatively long sequences. However, we believe that this is due to the random noise introduced in the read mapping process as well as the existence of primary RNAs. The correct conclusion should be implied by the Model-2 analysis, where the length effect on the expression levels of TE exons is negative for most samples.

**Figure 5:**
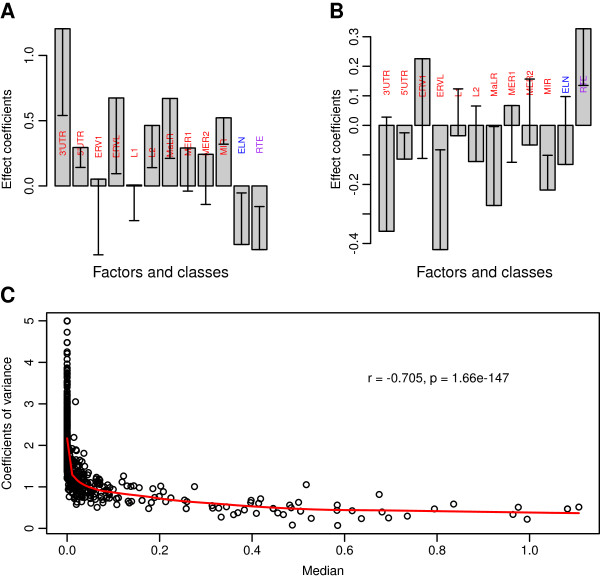
**The inverse relationship between the expression levels and expression variability of TE exons. A**: The effects of genomic factors on the middle digital expression level of TE exons (the median of the rescaled RPKMs across the 26 samples). **B**: The effects of genomic factors on the coefficient of variance for the rescaled RPKM of TE exons across the 26 samples. **C**: The scatter plot of the median(s) and the coefficient(s) of variance. The TE exons hosted by the genes un-expressed in over half of samples are excluded before the statistical analysis (using Model-2). The height of an error bar in Plots **A** and **B** represents the two-time standard error of the corresponding effect coefficient. In Plot **C**, the correlation was calculated by the Kendall method.

The high variability of the TE exon retention in transcripts prompted us to ask whether the genomic factors affecting the expression level of TE exons are also associated with the variability across the biological samples. In this regard, we repeated the linear model analysis with two descriptive statistics as the dependent variables. The two statistics are the median *(m)* and coefficient of variance *(v)* of a TE exon’s expression quantities across all samples. We calculated the *m* and *v* for each TE exon hosted by a gene expressed in at least 13 samples. The effect coefficients (see Methods section) of the defined continuous and categorical factors on the medians and coefficient of variance are visualized in Figure 5 (plots A and B). For the median, the effect coefficients of 3′UTR, 5′UTR, MIR, L2, MaLR, ELN and RTE are significant (p < 0.05), where the effect (coefficient) bars are longer than the corresponding error pikes. The profile is consistent with the sample-specific results presented in the previous paragraph. For the coefficient of variance, only the effects of 5′UTR, MIR and RTE are significant. However, as shown in plots A and B, the directions (positive or negative) of the most effect coefficients of the predictors for this variability measure *(v)* are reversed to those for the median *(m)*. Such a relationship is also reflected in the significantly negative correlation as demonstrated in the scatter plot of these two descriptive statistics (Figure 5C).

### An analysis on the biological function of TE exons

We considered a TE exon with the rescaled RPKM over 0.25 in at least two samples to be “highly expressed”. Accordingly, 327 genes hosting highly-expressed TE exon(s) were identified. Functional enrichment analysis using the GO Fat gene ontology functional annotation tool (available at DAVID [38]) showed that eight GO categories are over-represented by these genes at the threshold of False Discovery Rate (FDR) less than 0.05 (Table 3). The most significant term is molecular function (MF): zinc ion binding (FDR = 1.6-E4), where C2H2 ZNF genes amount over 60% of the total hits. ZNF genes represent one of the largest and most complex gene super-families in human genome, and are assumed to be the regulators of the expression of downstream genes [39]. The proteins of C2H2 ZNF genes contain zinc finger motifs of CX2-4CX3FX5LX2HX3-4HTGEKPYX (X represents any amino acid) forms. A recent publication reported that Alu-derived exons are preferentially established in this gene family [4]. We conjecture that such a preference may also hold for the exons derived from the DNA repeats of other TE families. Herein, we first generated the distribution table of the (ZNF) genes hosting the (highly expressed) TE exons across the cognate TE families. Then, by the Fisher’s exact test, we evaluated the null hypothesis (H_0_) that the classification (“highly expressed” or “other”) of exons derived from a specific TE family is independent of the host genes’ classification (“C2H2 ZNF” or “other”). TE families, including CR1, ERV1 and “other DNA” that didn’t contribute an exonized TE to ZNF genes according to the analyzed data, were excluded from this test. As shown in Table 4, with the p value less than 0.05, H_0_ was rejected in all major involved categories (TE families). This indicated that TE exons in ZNF genes are preferentially retained during the post-transcriptional modification, regardless of the family to which an exonized TE belongs. All C2H2 ZNF genes hosting the highly-expressed TE exons are summarized in Additional file 3.

**Table 3:** Functional enrichment analysis of the host genes with 327 highly expressed TE exons

**Category**	**Term**	**Gene number**	**P-value**	**FDR (%)**
MF^1^	GO:0008270 ~ zinc ion binding	59	1.16E-05	1.63E-02
MF	GO:0046914 ~ transition metal ion binding	66	3.01E-05	4.21E-02
MF	GO:0043169 ~ cation binding	85	3.18E-04	4.45E-01
MF	GO:0046872 ~ metal ion binding	84	3.93E-04	5.49E-01
MF	GO:0043167 ~ ion binding	85	5.37E-04	7.51E-01
BP ^2^	GO:0006350 ~ transcription	50	8.46E-04	1.35E + 00
MF	GO:0003677 ~ DNA binding	52	1.44E-03	2.00E + 00
BP	GO:0009101 ~ glycoprotein biosynthetic process	9	2.64E-03	4.17E + 00

^1^Molecular Function; ^2^Biological process.

**Table 4:** Classification and comparison of the C2H2 ZNF genes hosting highly-expressed TE exons

**TE**	**N1**	**N2**	**N3**	**N4**	**p**
Alu	850	51	143	17	7.65E-04
CR1	11	0	4	0	NA
ERV1	29	0	13	0	NA
ERVL	19	1	6	1	0.00E + 00
L1	148	12	24	5	4.71E-03
L2	79	7	34	5	2.27E-02
MaLR	57	1	18	1	0.00E + 00
MER1	40	2	13	1	1.00E-01
MER2	37	2	15	2	0.00E + 00
MIR	132	4	56	3	3.04E-02
Other_DNA	4	0	1	0	NA

**TE**: the categories of exonized TEs. **N1**: the number of the genes hosting TE exons. **N2**: the number of C2H2 ZNF genes hosting TE exons. **N3**: the number of genes hosting the highly-expressed TE exons. **N4**: the number of C2H2 ZNF genes hosting the highly-expressed TE exons. Among the 327 highly-expressed TE exons, 115 are included in the UCSC RefGene table.

## Discussion and conclusions

Computational identification of an exonized TE is usually based on the alignment with an exonic part of an EST (or cDNA) and the existence of canonical splice sites in the sequence. However, the functional importance of a TE exon is mainly determined by the expression level in a specific tissue and biological environment. In this study, we demonstrated that, with relatively high expression variability, most TE exons in mRNAs, especially those without exact counterparts in the UCSC RefSeq gene tables, demonstrate low but still detectable expression levels in most tissue samples. We also showed that the TE exons in CDSs are less likely to be constitutively expressed than those in 3′ (5′) UTRs. These results confirmed previous studies regarding the establishment of TE exons in the assembly of human mRNAs [4,21,22].

A recent study reported that MIR exons have highest inclusion levels among exonized TEs [23]. We found this result can be further generalized. That is, the exons derived from younger TE families (Alus, L1s) are less well established in the host genes than those derived from older TEs (MIRs, L2s) of the same super-classes, as indicated by the expression quantities including the measures of presence, level and variability. A deviation from the general association is that, although the activity burst of L1 families was 20–30 million years earlier than that of Alus [40,41], the expression quantities of the exons derived from the elements of these two families are similar to each other (p & 0.05). This similarity may be due to their common consensus target sites [42,43]. Another explanation is that the insertion of Alu and L1 elements frequently disturbs the stability of the target gene [14], and the human genome evolution process has likely formed a mechanism to remove the exons derived from such elements.

A previous study showed that the lengths of exons inversely influenced the TE inclusion levels in transcripts [44]. This relationship was confirmed by our analysis focused on exonized TEs. We are especially interested in this finding and wonder if it can be also derived by EST data analysis. Thus, we repeated the linear statistical analysis using EST-based inclusion level (retrieved from the TranspoGene/Human exonized Tes table) as the dependent variable in the place of rescaled RPKM. The result moderately supports the RNA-seq data based analysis in that the observed length effect is negative but not significant (p & 0.05). Such an observation even holds when the analysis is focused on the subset of Alu-derived exons. Herein, we speculated that, to which extent, the digital expression quantities of TE exons are consistent with the inclusion levels calculated using EST data [21]. By a simple regression analysis (Additional file 4), we found that 47.8% of variability across TE exons in the digital expression measure can be explained by the inclusion levels (p = 0). These results indicate that the EST data and digital expression data can be complementary in inferring the expression patterns of TE exons.

Our work also extended the previous studies by several novel findings. Among them, the finding that a high TE nucleotide proportion leads to lower retention rates of the expressed TE exons in mRNAs is intuitively correct. The other two as described below warrant further investigation.

First, the considered genomic factors impact the expression level and the expression variability of TE exons in inverse ways. For examples, compared to those derived from Alus, MIR-derived exons have high rescaled RPKMs but the variability measure (CV) across the samples is lower. The expression profile of MIR exons suggests that they may have become functionally essential in human.

Second, not only the exons derived from Alu elements but also the exons from the transposons (retrotransposons) of other families were preferentially established in C2H2 ZNF genes. In fact, such a preference is more remarkable in the exons derived from ancient TEs. For example, among the seven TE exons derived from L2 elements and hosted by ZNF genes, five are highly expressed. However, because most TE exons are derived from Alu elements, the roles of these elements in shaping ZNF genes and in the regulatory functions are most predominant.

Understanding the expression pattern of the TE exons not annotated in RefSeq is of special biological importance. For these exons, the distribution of rescaled RPKMs shown in Figure 1 (represents the 16 sample in cluster G1) can be regarded as a canonical profile. Herein, a challenging question is why the reads mapped to the genome regions are so rare to detect in the samples of cluster G2 (Additional file 1), which include three prostate tumors (prAd-1, 2, 3) and one normal prostate sample (prNorm-3). In the Results section, we explored this issue from biological aspects. Another potential explanation lies in the filtering process of the mapped reads. This can be scrutinized from two aspects. First, a relatively short read naturally has more chances to be aligned to multiple sites of the reference genome and thus to be filtered out as an “ambiguously mapped read”. Second, TE exon sourced reads are more likely to be “repetitively mapped” compared to others. As a result, the true expression level or un-expression ratio of TE exons may be underestimated or overestimated in G2 samples where the read length is short (33 bp). However, this elucidation is not supported by the information of two prostate (cancer) cell lines. That is, the samples LNCaP and PrEC have the read length (35 bp) close to G2 samples but the expression profile of TE exons similar to G1 samples. Apparently, this phenomenon warrants additional follow-up in independent laboratory studies.

As discussed above, the assembly of short sequencing reads derived from repetitive elements is potentially associated with a high unmapped fraction. The problem arises from exclusion of ambiguously mapped reads in data preprocessing, and is likely serious for a dataset generated by single-read sequencing methods. However, for a dataset generated by paired-end sequencing methods, in which the two ends of an mRNA fragment are measured, the uncertainty should be largely alleviated [45]. A plausible explanation is that, in mapping paired-end reads, the hit(s) of a read is determined not only by its alignment with the genome but also by the alignment of its mate read [46]. Usually, an mRNA fragment to be measured in paired-end RNA-seq experiments is long (at least 200 bp) [33,35], sufficient to cover a sequence longer than a medium-sized exon. In this context, the probability that a paired read is mapped to multiple genomic positions is low. The observed repetitive hits can be largely attributed to the existence of paralogous genes. Accordingly, we can assume the chances for ambiguously mapped reads solely due to repetitive elements are rare in paired-end RNA-seq datasets.

It should be noted that in the linear model analysis, we included four independent variables whose quantities or categories can be directly inferred from the TranspoGene human exonized TEs table as the predictors of the retention level of TE exons. We believe that the quality of the splice sites of the exonized TEs is a promising predictor for their retention levels in mature mRNAs. However, the measurement of splice quality is yet an unsolved challenge, though many studies on the prediction of splice sites have been published [44,47]. We have not devised an appropriate method to derive a measure that is related to the sequences of the splice sites and is in association with the retention levels. Solving this challenge is the goal of another study in our research agenda.

In this study, we employed a ratio quantity, i.e. the rescaled RPKM (see Methods Section), to infer the expression (retention) level of an exon. Theoretically, this estimate may be biased due to the potential existence of primary RNAs. However, due to following reasons, it does not impact the validity of our conclusion on the variability and influential factors for the TE exon retention. First, the reads mapped on the simulated exons, i.e. the intron sequences immediately downstream of the un-annotated TE exons, are very rare in the analyzed datasets except for the six ovarian cell line samples. Second, we used the rescaled RPKM of the simulated exons as the baseline to determine the “expressed” genes and to test the existence of the transcripts containing un-annotated TE exons. An alternative quantity (in the place of rescaled RPKM) is the inclusion level for an (TE) exon of interest. The value can be computed from the counts of the reads mapped to the flanking exon-exon junctions [6]. However, in this way, most reads in a dataset will be unused because the length of a junction must be shorter than the read length. As a result, given the limited sequencing depth of currently published RNA-seq datasets for individual samples, the inclusion level estimate of an alternative exon may be severely biased or difficult to explain because of the unbalanced read counts in flanking junctions as shown in the Figure 1 of [4]. Therefore, there is a dilemma on using either of the two methods to exactly model the splicing pattern of a specific exon, but this is beyond the scope of our study.

As mentioned in the Results section, we hypothesize that there is a tissue-specific mechanism underlying the association between the expression-based sample grouping (G1, G2 and G3) and the tissue/disease type-based classification. However, so far, we lack additional data to provide further evidence for this hypothesis. On the other hand, the differences among G1, G2 and G3 in the profiles of the retention rates of TE exons could be attributed to the variations of the used experimental protocols (especially those for cell culture) in generating the analyzed data. Previous studies have shown that pol III SINE transcripts increase when the cells are stressed [27,38]. However, it seems that this finding cannot explain our observation. More specifically, many TE exons (including those derived from SINEs) demonstrate high inclusion levels in the six ovarian cell lines (G3), but the G3 cells were not stressed, i.e. they were cultured in normal medium and temperature [36]. Another related problem is that substantial pre-RNAs are observed present in the six ovarian cell lines (G3 class) but not in other samples (Figure 1, Additional files 1 and 2). One may speculate whether the variation of methods for extracting mRNAs may cause such a difference. We compared the experimental protocols of the 26 RNA-seq datasets listed in Table 1, and found that the method [36] used to extract mRNAs from G3 samples is the same as the method (using Invitrogen TRIzol) [32] employed for the liver tissue samples (of G1 class). This indicates that there could be other factors contributing to this issue.

A recent publication showed that Alu exonization is controlled by the competition of hnRNP C and U2AF65 genes [39]. We found that the RPKMs of hnRNP C gene in G2 and G3 samples are significantly (p < 0.01) lower than those in G1, while such a group specific characteristic is not observed for the expression level of U2AF65 gene (p & 0.05). This observation, together with the descending order of the digital expression levels of TE exons from G3, G1 to G2 modestly suggests that hnRNP C (but not U2AF65) regulate the exonization of TEs in a non-linear way. In other words, this implies that both too high (G3) and too low (G2) exonization levels of TEs are related to a lower expression level of hnRNP C gene.

## Methods

### RNA-seq data preprocessing

We downloaded the RNA-seq read data from the NCBI SRA database (Table 1) [36]. The TopHat software [46] was employed to map the short reads onto the human genome (hg19) and the computationally identified exon-exon junctions. In the execution, we set “anchor length” as 4, and “--segment-length” as half of the read length. “Mate-inner-dist” (for paired-end data) was estimated by the difference between the middle RNA fragment length and twice the read length. Other parameters were set as default in TopHat (v-1.3.2) including two mismatches allowed for a read [49]. We divided the output file of accepted hits in SAM format into a set of relatively small files with each corresponding to a chromosome. Meanwhile, we filtered out the ambiguously mapped reads that have non-primary alignments and repetitive hits. A lab-owned R program was then used to compute the digital gene expression profiling. More specifically, by referring to the UCSC RefSeq gene tables, we first counted the number of reads mapped to the region of an exon that resides in at least on RefSeq gene. Then, the RPKM for an exon was calculated using the method mentioned in [50] (Additional file 5). After that, the expression level of a RefSeq gene was determined by averaging the RPKMs of the contained exons (Additional file 6). Finally, we calculated the rescaled RPKM of an exon by dividing its RPKM with the expression level of its host gene. The rescaled RPKM of a TE exon un-annotated to any RefSeq gene or of a simulated exon (see the Results section) was determined using the same method but its RPKM was excluded from calculating the gene-level expression. Other data preprocessing procedure includes the threshold treatment of rescaled RPKMs with an upper limit of 10 in the subsequent analysis.

### Exonized TE (TE exon) data

We downloaded Table Human Exonized TEs from the TranspoGene website [21]. Genome coordinates were converted from hg18 (Human Genome version 18) to hg19 by the LiftOver tool hosted on the UCSC genome browser server [37]. The location in the host gene, length (ELN), cognate TE family, TE nucleotide proportion (RTE) and EST-based inclusion level of a TE exon were directly retrieved or derived from this table. In particular, RTE was calculated as the proportion of the nucleotides originated from the cognate TE in the sequence of a TE exon.

### C2H2 ZNF genes

We extracted (11/05/2011) the C2H2 ZNF gene list from the DAVID Bioinformatics Resources [48], where the collection of these genes was based on the protein domain annotation resource SMART [51].

### Two-step linear analysis

To investigate the effects of genomic factors on the expression of TE exons, we adopted a two-step linear model analysis approach similar to [15]. The motivation is that a single linear model is not sufficient to analyze the observed data where a substantial proportion (e.g. approximately 30-80% in different datasets) of TE exons have no RNA-seq reads mapped to the genomic regions and, as a result, we cannot conduct the logarithmic transformation of the expression levels (rescaled RPKMs) to resemble a normal distribution. The proposed method consists of a logistic regression model and a simple linear model. Below are the mathematical formulations of these two models.

$$\;\begin{array}{ll} \mathrm{Model}-1: & \log\left(\frac{P\left(z_{i}=1\right)}{1-P\left(z_{i}=0\right)}\right)\\ & =\mu +R_{i}\alpha +F_{i}\beta +\gamma l_{i}+\phi c_{i}\\ \mathrm{Model}-2: & \log\left(y_{j}\right)\\ & =\mu^{*}+R_{j}\alpha^{*}+F_{j}\beta^{*}+\gamma^{*}l_{j}+\phi^{*}c_{j}+e_{j}\\ & \;\left(y_{j}\&0\right)\; \end{array}$$

In Model-1, *z*_*i*_ ∈ {1, 0} indicates if exon *i* has at least one read mapped onto the genomic region. *R*_*i*_ and *F*_*i*_ are a three-element row indicator vector for the location (in the host gene) category (CDS, 3′UTR, or 5′′UTR) and a nine-element row indicator vector for TE category (Alu, ERV1, ERVL, L1, L2, MaLR, MER1, MER2, or MIR) of the exon, respectively. *l*_*i*_ is the log10 transformed sequence length (ELN) and *c*_*i*_ is the TE nucleotide proportion (RTE) of the exon. In Model-2, for TE exon *j, y*_*j*_*and e*_*j*_ are the normalized expression level and random noise, respectively. *R*_*j*_, *F*_*j*_ , *l*_*j*_, and *c*_*j*_ are similarly defined as in Model-1. (*μ*, **α**, **β**, *γ*, *ϕ*) and (μ*, α*, β*, γ*, ϕ*) are the parameter sets of the two models to be estimated. Among them, **α** (α*) and **β** (β*) are three-element and nine-element column vectors, respectively, and others are scalars. The estimates of these parameters are called effect coefficients. Model-1 evaluates the effects of genomic factors on the presence or absence of TE exons in mature transcripts. Model-2 evaluates the effects of the genomic factors on the expression levels of the TE exons with non-zero RPKMs. We performed the logistic regression analysis using the procedure *lrm* included in the R package “Design”. The simple regression analysis was conducted with the procedure *lm* in the R package “stats”. In the implementation, we set CDS as the baseline for the location factor, and Alu as the baseline for the TE family factor. Therefore, the first element of **α** (α*) and **β** (β*), was set as zero in both models.

## Competing interests

The authors declare that they have no competing interests.

## Authors’ contributions

WZ and KZ conceived and designed the experiments, performed the statistical analysis and drafted the manuscript. AE, WF, PD and ZF helped in the experimental design and participated in writing. KZ supervised and coordinated the project. All authors read and approved the final manuscript.

## Supplementary Material

Additional file 1 **Histogram for the digital expression levels of TE exons in sample prAd_1 (representing cluster G2).** The black bar on the left side of each plot represents the proportion of un-expressed exons. Click here for file

Additional file 2 **Histogram for the digital expression levels of TE exons in sample OV-1-pr (representing cluster G3).** The black bar on the left side of each plot represents the proportion of un-expressed exons. Click here for file

Additional file 3 C2H2 ZNF genes hosting the highly-expressed TE exons. Click here for file

Additional file 4 **The positive correlation between the middle digital expression quantities of TE exons in the 26 samples and the inclusion levels calculated using the EST data**[21]. Click here for file

Additional file 5 Raw digital expression levels of TE exons (RPKM). Click here for file

Additional file 6 Rescaled RPKMs of TE exons. Click here for file
