# Supplementary figures and images for: Inferring the expression variability of human transposable element-derived exons by linear model analysis of deep RNA sequencing data
Source: BMC Genomics. 2013 Aug 28;14:584. doi: 10.1186/1471-2164-14-584 (PMC3765721; doi:10.1186/1471-2164-14-584)

**Constitutive exons**

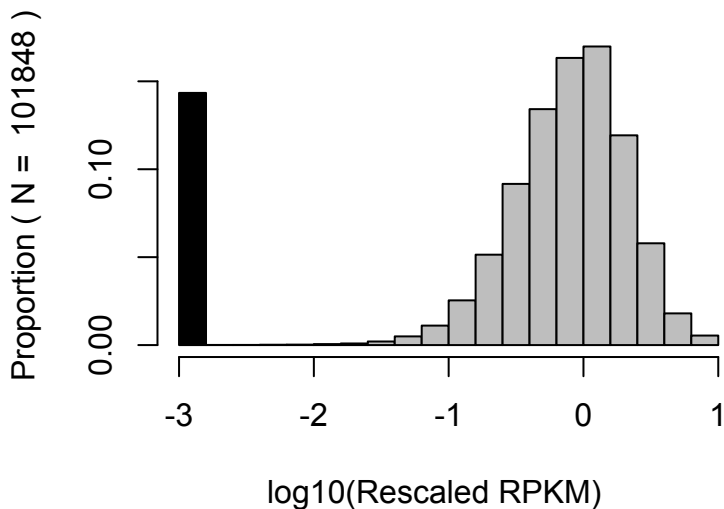

**Cassette exons**

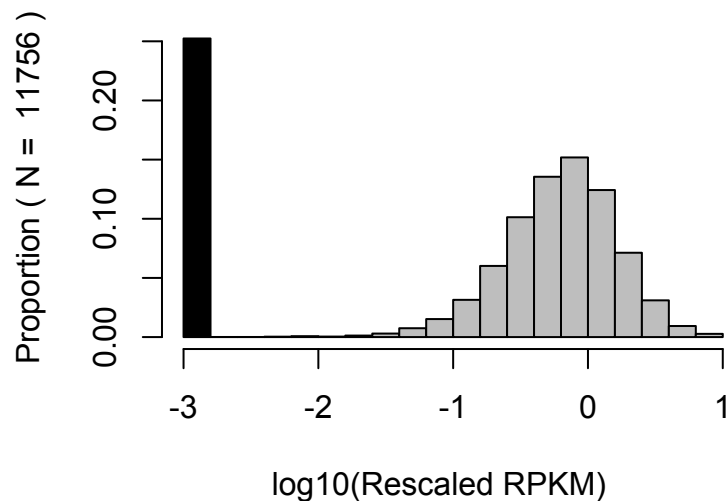

**Annotated TE exons**

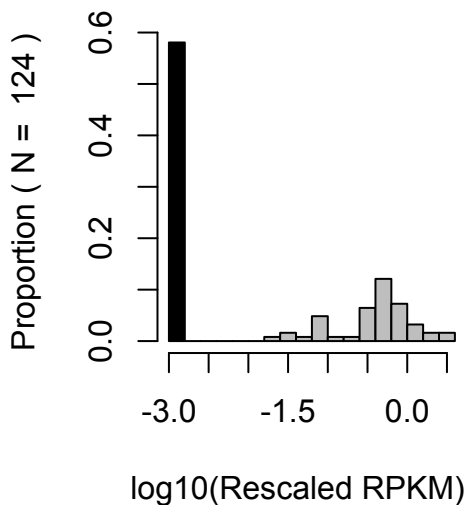

**Un-annotated TE exons**

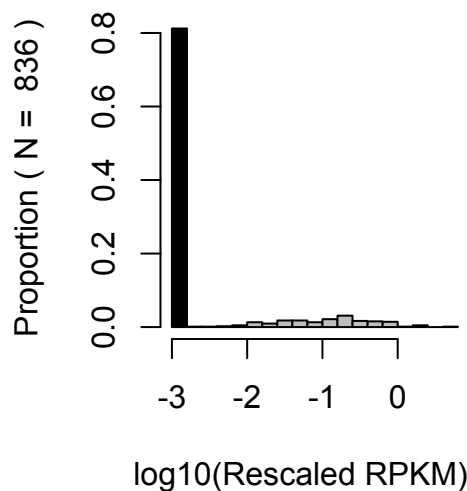

**Simulated exons**

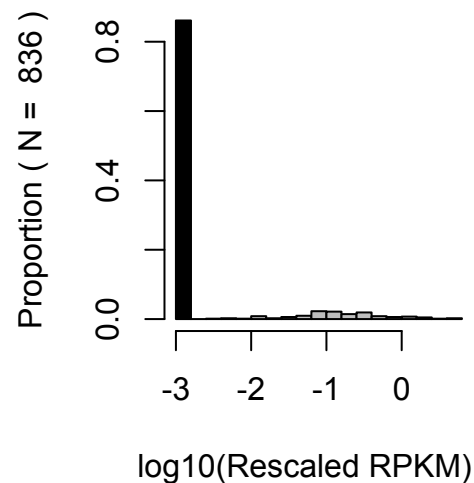

Supplement: Additional file 1 — Histogram for the digital expression levels of TE exons in sample prAd_1 (representing cluster G2). The black bar on the left side of each plot represents the proportion of un-expressed exons. [file 1471-2164-14-584-S1.pdf]

**Constitutive exons**

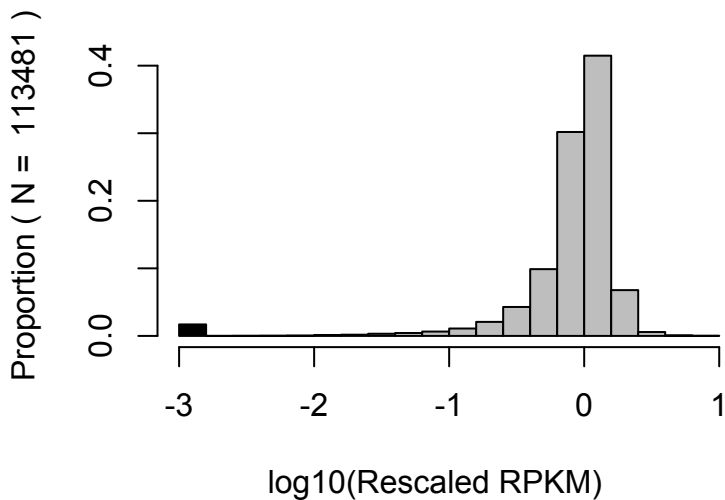

**Cassette exons**

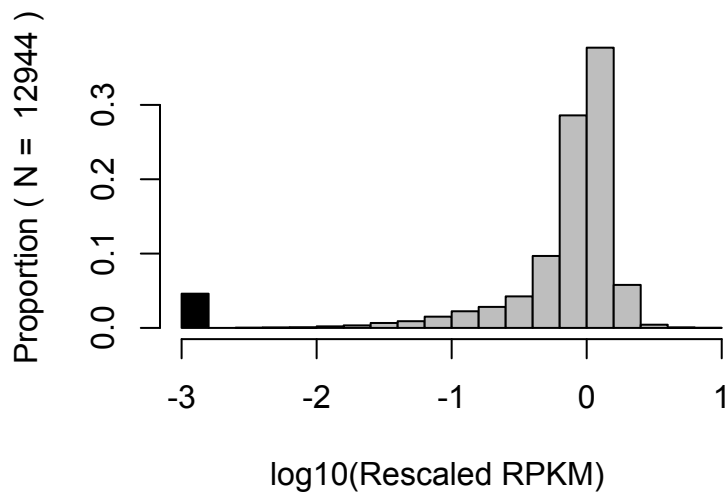

**Annotated TE exons**

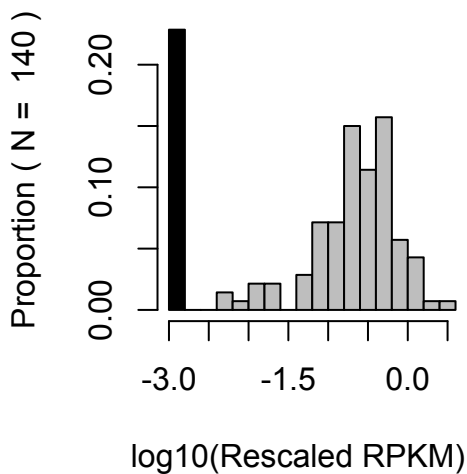

**Un-annotated TE exons**

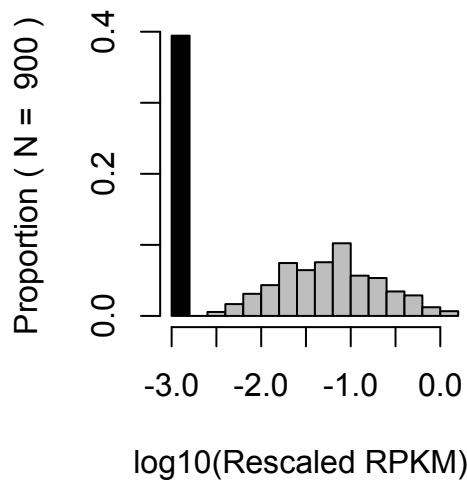

**Simulated exons**

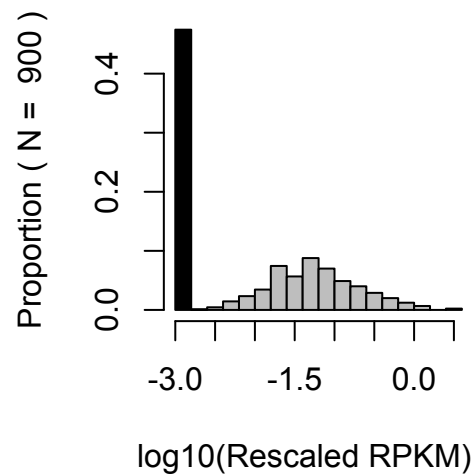

Supplement: Additional file 2 — Histogram for the digital expression levels of TE exons in sample OV-1-pr (representing cluster G3). The black bar on the left side of each plot represents the proportion of un-expressed exons. [file 1471-2164-14-584-S2.pdf]

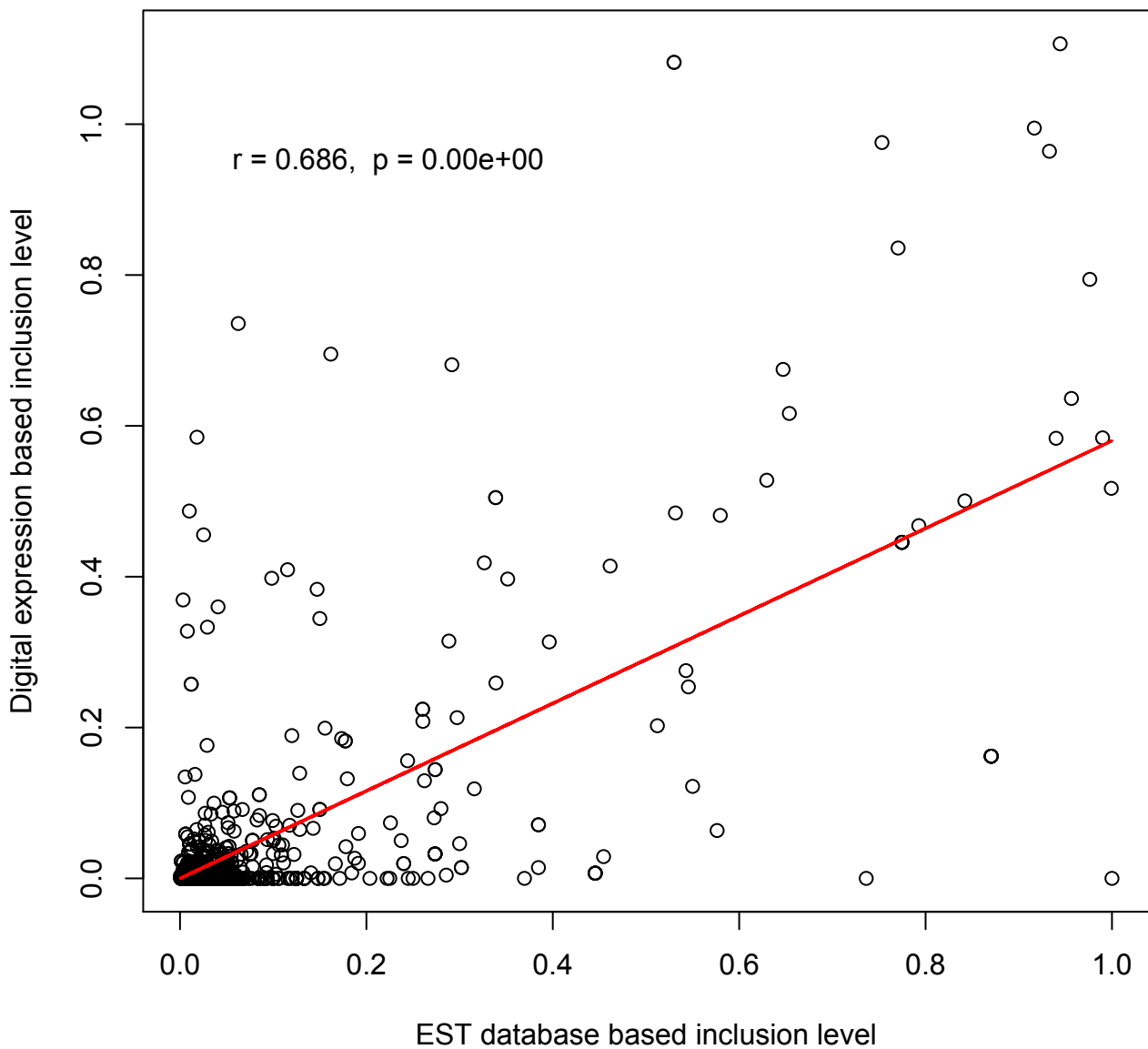

Supplement: Additional file 4 — The positive correlation between the middle digital expression quantities of TE exons in the 26 samples and the inclusion levels calculated using the EST data[21]. [file 1471-2164-14-584-S4.pdf]
